# Supplementary material for: Insights from the Biorepository and Integrative Genomics pediatric resource
Source: Nat Commun. 2025 May 22;16:4750. doi: 10.1038/s41467-025-59375-0 (PMC12098674; doi:10.1038/s41467-025-59375-0)
Supplement: Supplementary file 1 — Supplementary Information [file 41467_2025_59375_MOESM1_ESM.pdf]

10 Supplementary Figures

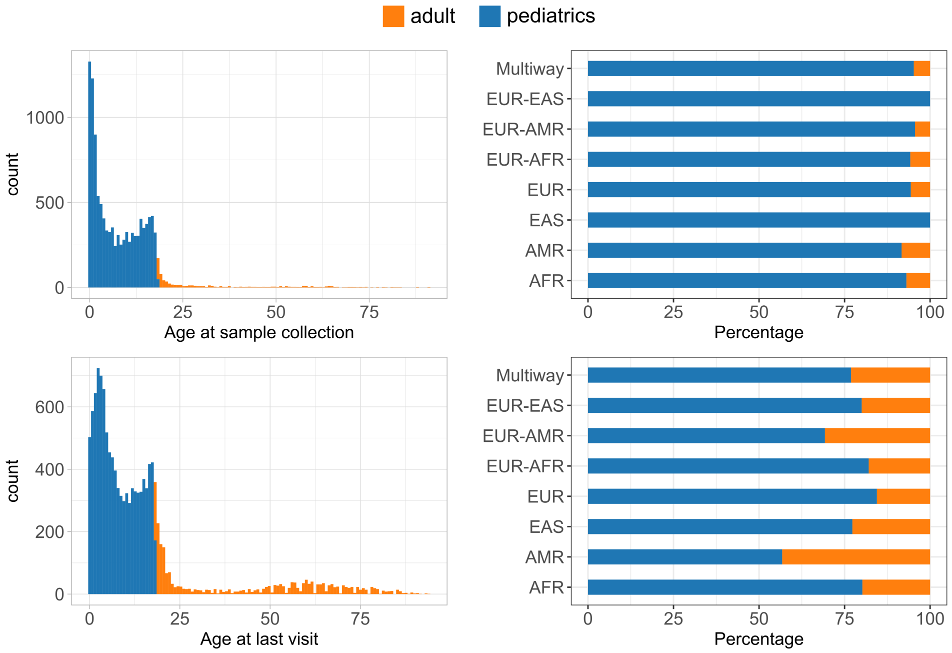

Supplementary Figure 1: **Age distribution.** Distribution of age at sample collection (top) and at the last visit (bottom). The right panels: bar plots illustrating the percentage distribution of demographic categories, stratified by inferred ancestry.

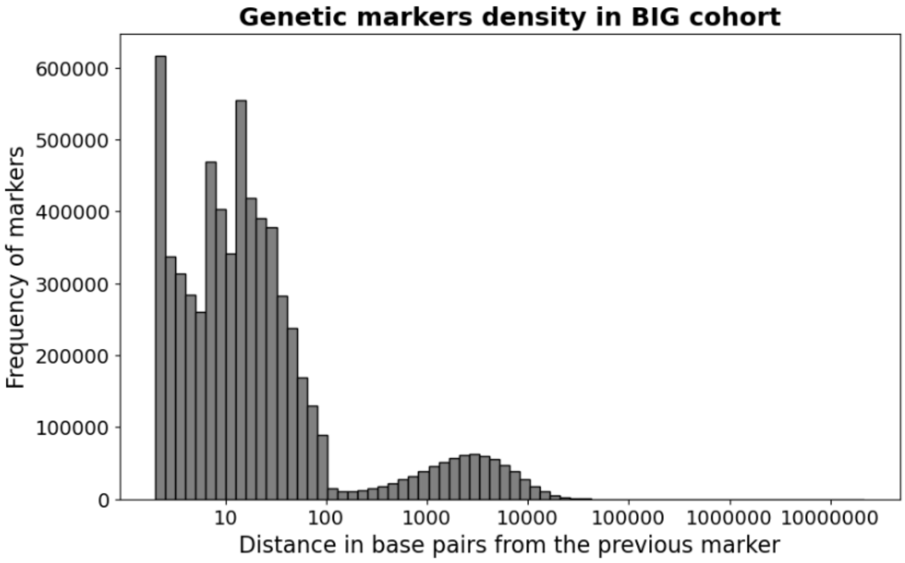

Supplementary Figure 2: **Distance between consecutive variable sites.** Distribution of distances for all pair of variants. The distribution is multimodal, roughly corresponding to modal distance for markers in the coding (lower modes) and non-coding (higher mode) regions. Overall, the markers appear to be evenly distributed, indicating good coverage across the entire genome.

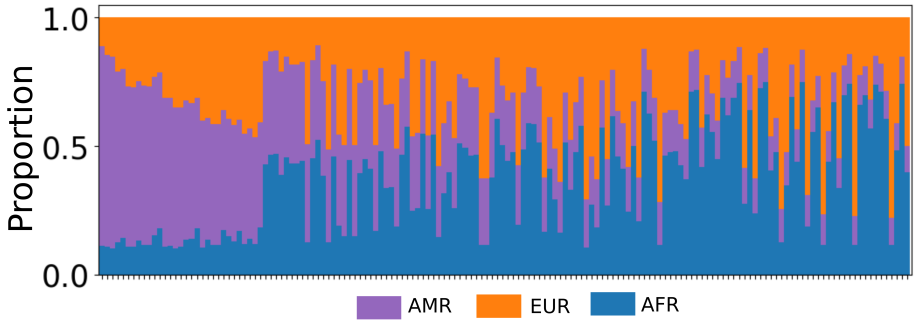

Supplementary Figure 3: **Details of the ancestry components in multi-way admixture.** To facilitate proper visualization of the patterns of admixture, we provide here a magnification of part of **Figure 1b**

.

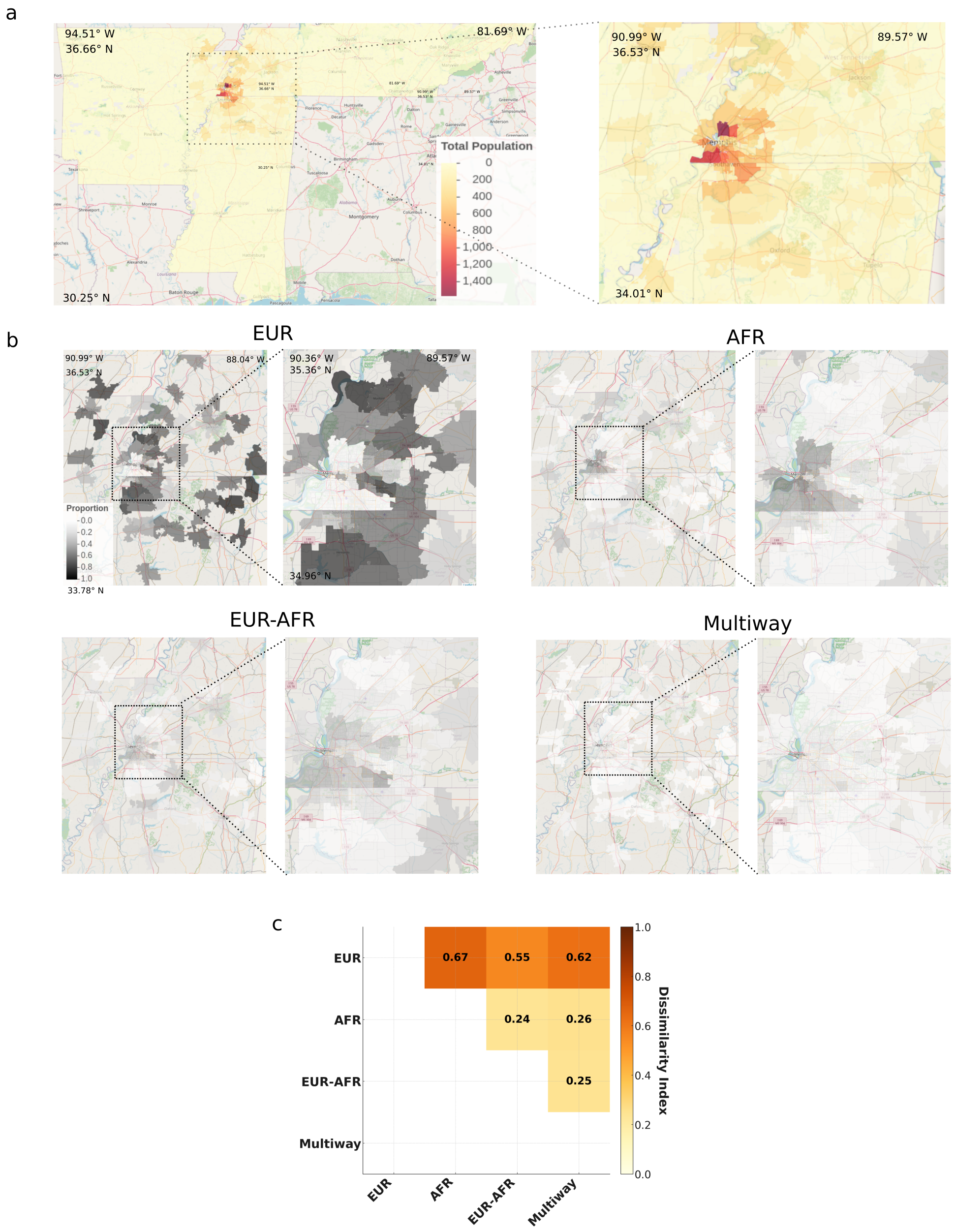

Supplementary Figure 4: **Demography of enrolled participants.** **(a)** Number of enrolled participants by ZIP code. The region surrounding Memphis City is zoomed in. **(b)** Proportion of individuals by zip code for inferred ancestries with more than 500 individuals. **(c)** Pairwise Dissimilarity index between ancestries, considering the proportion in each zip code. The dissimilarity index measures the extent of segregation between two groups across geographic areas, indicating the proportion of one group that would need to relocate to achieve an even distribution relative to the other group, with values ranging from 0 (perfect integration) to 1 (complete segregation). Maps were produced with the `leaflet` package (v. 2.2.1) using GeoJSON data for state ZIP-code boundaries publicly available.

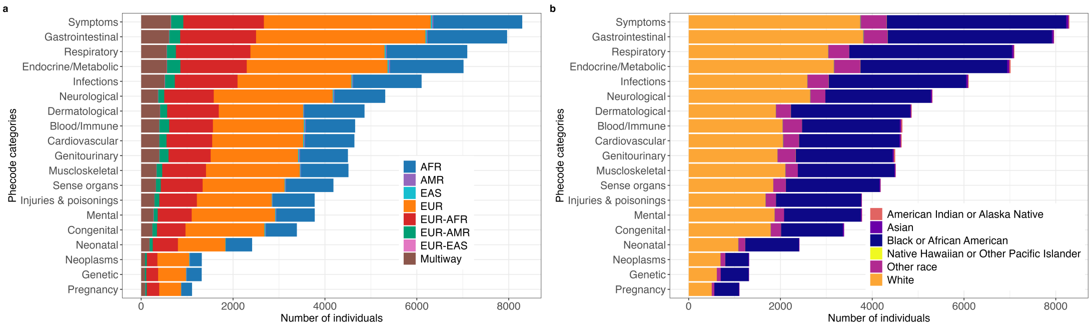

Supplementary Figure 5: **Phenotypes prevalence in electronic health records in participants with sequence data** stratified by inferred ancestry **(a)** and self-reported race **(b)**. Phenotypes are grouped into Phecode categories. Distribution of ancestries in Phecode categories reflect the global distribution of ancestry

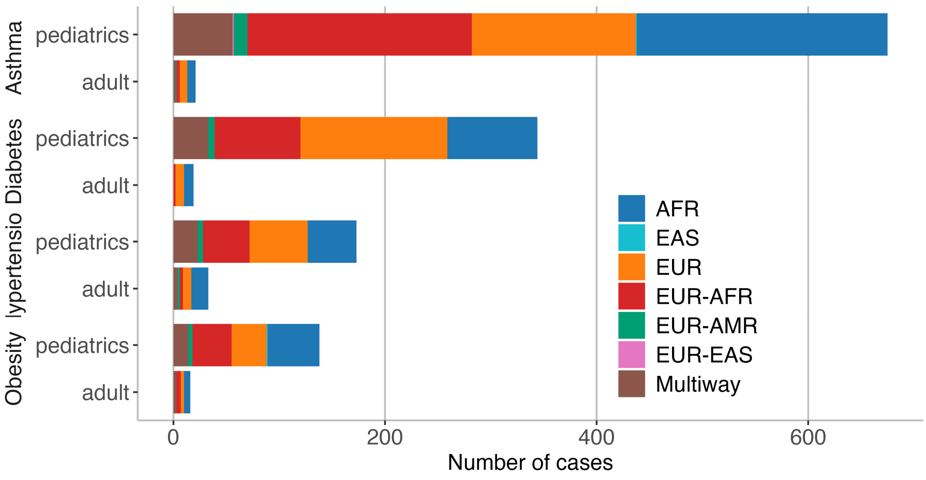

Supplementary Figure 6: **Count of cases across Asthma, Diabetes, Hypertension, and Obesity**. Cases are categorized by pediatric and adult populations and color-coded by inferred ancestry groups: AFR (African), EAS (East Asian), EUR (European), EUR-AFR (European-African), EUR-AMR (European-American), EUR-EAS (European-East Asian), and Multiway.

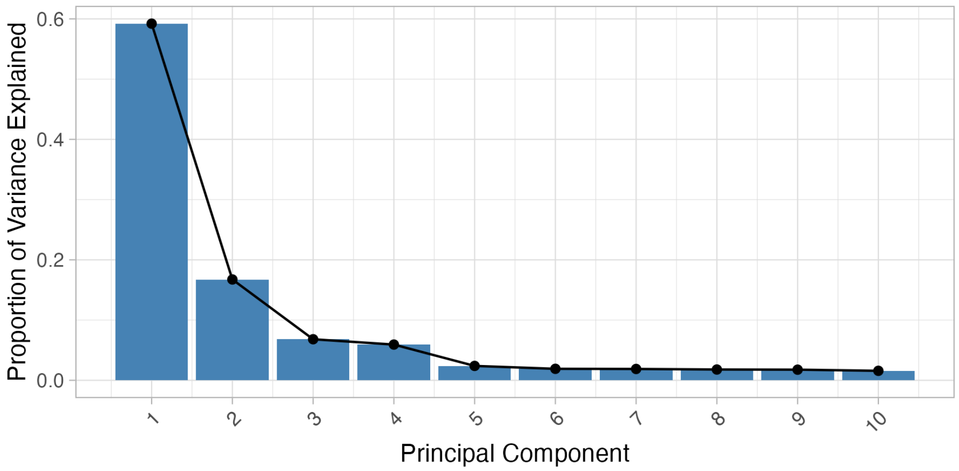

Supplementary Figure 7: **Scree plot for Principal Component Analysis in figure 3a** . Only the first ten components are reported. The first two components explain 76% of the variance.

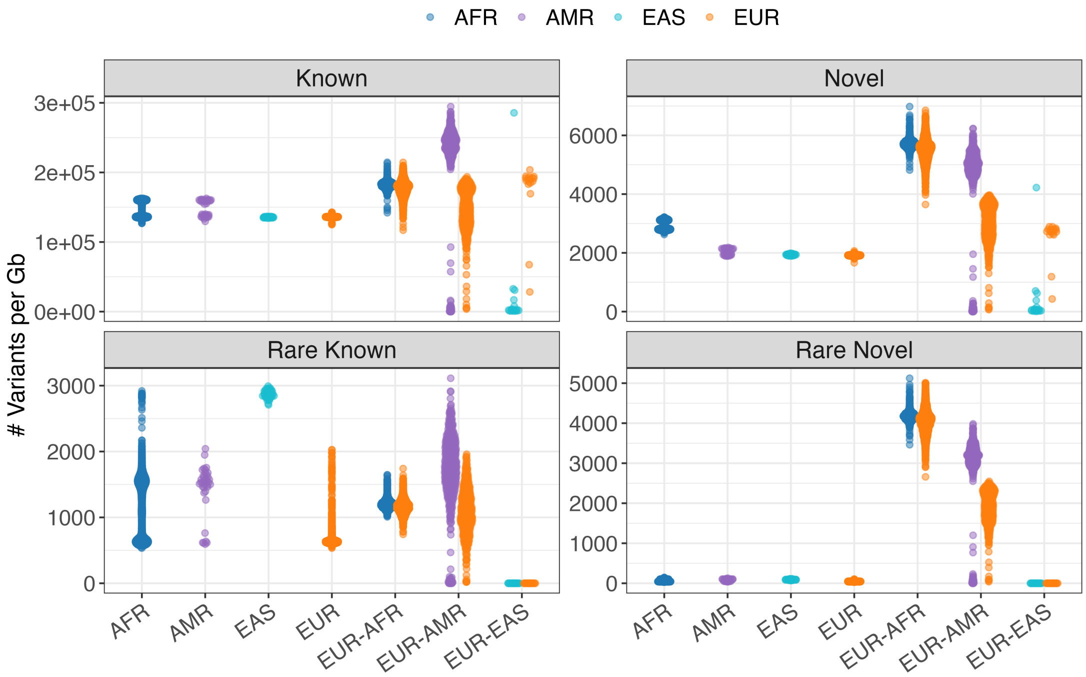

Supplementary Figure 8: **Distribution of known and novel variants across ancestral backgrounds.** Violin plots show the density distribution of variants per gigabase (Gb) across different ancestry groups. Top panels show all variants while bottom panels show rare variants (alternate allele frequency $<1\%$). Left panels display known variants (present in reference databases) and right panels show novel variants (absent from major databases like gnomAD, 1000 Genomes, and HGDP). Ancestry categories include African (AFR), American (AMR), East Asian (EAS), European (EUR), and admixed groups (EUR-AFR, EUR-AMR, EUR-EAS). Note the elevated number of novel variants in admixed populations, particularly in EUR-AFR and EUR-AMR groups.

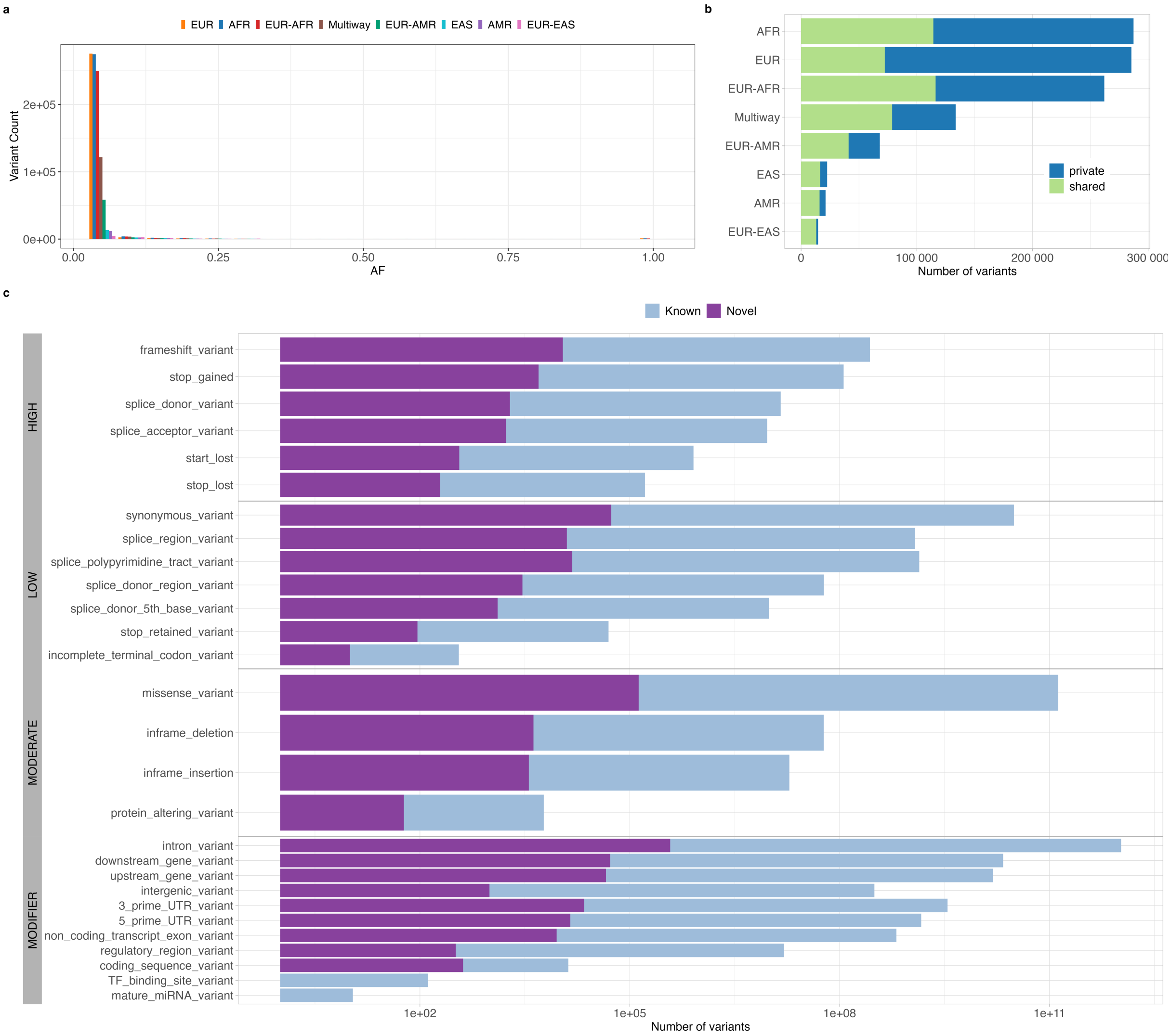

Supplementary Figure 9: **Features of novel variants.** **(a)** Allele frequency spectrum showing the prevalence of rare variants. **(b)** Counts of variants by ancestry stratified by private and shared with another one or more ancestries. **(c)** Counts of variants (log scale) by annotated consequences for novel and known variants.

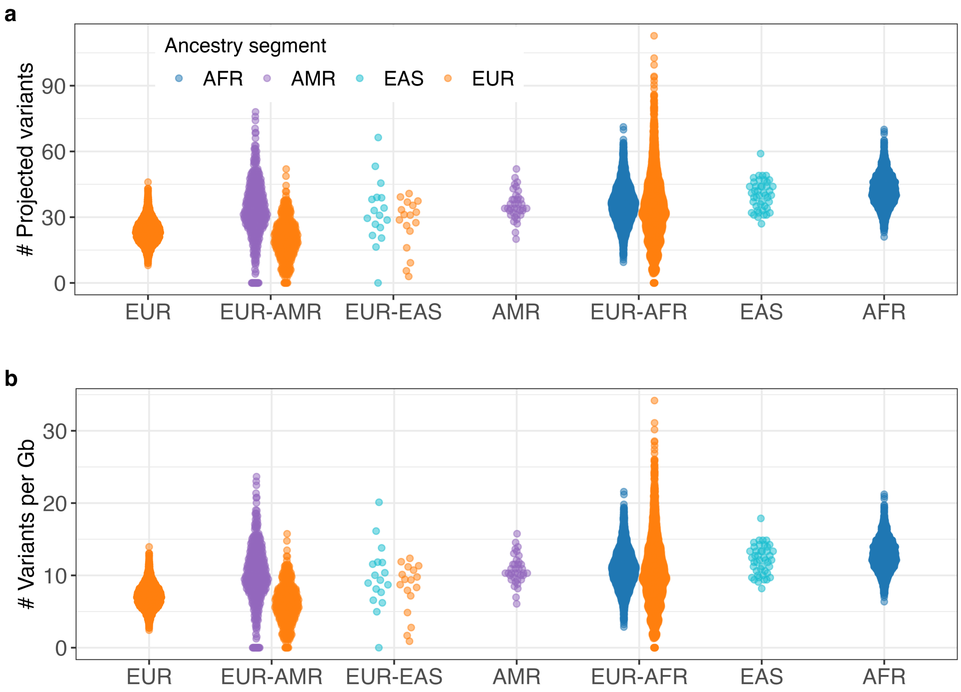

Supplementary Figure 10: **Counts per individual of rare deleterious variant by ancestry.** Rare deleterious variants are defined as having alternate allele frequency <1% in the total BIG samples, and classified as high impact or missense with SIFT<0.05 and Polyphen>0.85. Variants counts take into account the inferred ancestry of the genomic tract in which they are located, therefore individuals in admixed groups are represented twice. In panel **(a)** counts per ancestry tract are normalized by the proportion of ancestry and therefore the y-axis represent the projection as the ancestry tract was as long as the whole genome. In panel **(b)** we report counts per Gb. Ancestry categories include African (AFR), American (AMR), East Asian (EAS), European (EUR), and admixed groups (EUR-AFR, EUR-AMR, EUR-EAS).

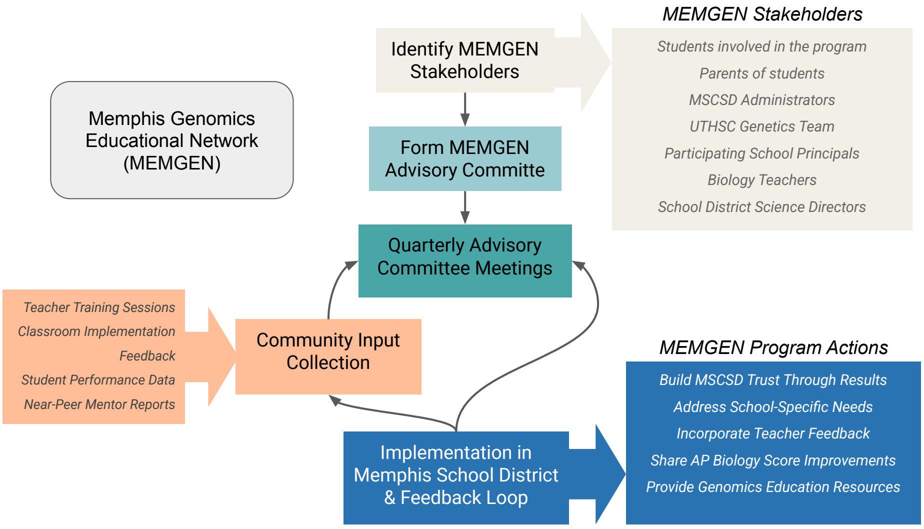

Supplementary Figure 11: **Flow diagram illustrating the organizational structure and feedback processes of the Memphis Genomics Educational Network (MEMGEN)**. The diagram shows how stakeholder input from Memphis Shelby County School District (MSCSD) administrators, teachers, and genetics experts feeds into program implementation through an advisory committee system, with continuous feedback loops ensuring program improvement and effectiveness in genomics education delivery across Memphis schools.

11 Supplementary Tables

Supplementary Table 1: **Examples of large pediatric cohorts.** Although the list is not exhaustive, it is intended to provide context for understanding BIG's position in terms of size, diversity, and data availability.

Reported Ancestry Representation	Cohort Name	Size	Start Date	Reported Ancestry	Study Group Type	EHR Availability	Genetic Data
Predominantly one ancestry	Avon Longitudinal Study of Parents and Children (ALSPAC) [36]	14,000 children	1991	Predominantly European descent, reflecting the population of the Avon area in the UK	Mother-Child	Has EHR	Has genetic data
	Copenhagen Prospective Studies on Asthma in Childhood (COPSAC) [86]	700 children	1998	Primarily Danish, reflecting the population of Denmark	Children Only	No EHR	Has genetic data
	The Norwegian Mother and Child Cohort Study (MoBa) [37]	114,500 children	1999	Predominantly Norwegian, reflecting the population of Norway	Mother-Child	Has EHR	Has genetic data
	Longitudinal Study of Australian Children (LSAC) [87]	10,000 children	2004	Predominantly Australian, with representation from various ethnic backgrounds	Children Only	No EHR	No genetic data
	All Our Families (AOF) Cohort [38]	3,000 families	2008	Primarily of European descent, reflecting the population of Calgary, Canada	Mother-Child	Has EHR	No genetic data
Diverse ancestries	Children of Philadelphia (CHOP)	100,000 children	2006	Diverse, reflecting the population of Philadelphia	Mother-Child	Yes	Has genetic data
	Childhood Cancer Survivor Study (CCSS) [29]	24,000 survivors	1994	Diverse, reflecting the population of North America	Children Only	Has EHR	Has genetic data
	The Boston Birth Cohort [30]	8,000 births	1998	Predominantly African American and Hispanic participants	Mother-Child	Has EHR	Has genetic data
	Generation R Study [31]	10,000 children	2002	Multi-ethnic urban population, including Dutch, Surinamese, Turkish, Moroccan, and others	Mother-Child	Has EHR	Has genetic data
	Pediatric Imaging, Neurocognition, and Genetics (PING) Study [32]	1,400 children	2009	Diverse, including African American, Asian, Hispanic, and non-Hispanic White participants	Children Only	No EHR	Has genetic data
	NICHD Fetal Growth Studies [33]	2,400 pregnancies	2009	Diverse, including African American, Asian, Hispanic, and non-Hispanic White participants	Mother-Child	Has EHR	Has genetic data
	Biorepository for Integrative Genomics (BIG) [28]	42,000	-	Diverse, including African American, Asian, Hispanic, and non-Hispanic White participants	Children Only	Has EHR	Has genetic data
	Healthy Brain Network (HBN) [34]	10,000 children	2015	Diverse, with efforts to include underrepresented populations	Children Only	No EHR	Has genetic data
	Environmental Influences on Child Health Outcomes (ECHO) [35]	50,000 children	2016	Diverse, with efforts to include underrepresented populations	Mother-Child	Has EHR	Has genetic data

Supplementary Table 2: **Simplification of self-reported race entries in electronic health records.** The purpose of mapping self-reported race to standardized categories is to simplify the analyses and eliminate the use of inaccurate or inappropriate terminology [HL7 v2.5 race classification]

[88].

**Self-Reported Race**	**Number of individuals in self-reported race**	**Standardized HL7 v2.5 Race Category**	**Number of individuals in Standardized HL7 v2.5 Race Category**
Black or African American	4905	Black or African American	5297
African American	392		
White	5672	White	6115
White or Caucasian	89		
Caucasian	354		
Amer. Indian/Alaska Native	8	American Indian or Alaska Native	35
Native American	27		
Asian	68	Asian	69
Asian or Pacific Islander	1		
Native Hawaiian/Other Pacific Islander	4	Native Hawaiian or Other Pacific Islander	6
Pacific Islander	2		
Other	14	Other race	922
Other/Unknown	410		
Amharic (East African)	1		
East Indian	2		
Hispanic	268		
Middle Eastern	3		
Multiple	224	Unknown race	45
Unavailable	37		
Declined	4		
Patient Declined to Answer	3		

Supplementary Table 3: Details of the logistic regression analysis in Figure 3D, relative to the incidence of novel variants by category of variant's impact. The model estimates log-odds of a variant being novel (dependent variable) based on its impact category (independent variable), with MODIFIER impact serving as the reference level. Estimates represent the change in log-odds associated with each impact category relative to MODIFIER variants. All p-values are from two-sided tests with no adjustments for multiple comparisons

Term	Estimate	Std. error	Statistic	p.value	conf.low
Intercept	-2.09	0.001	-1430	$< 2e-16$	-2.10
HIGH	0.95	0.008	116	$< 2e-16$	0.93
LOW	-0.118	0.004	-30.3	$< 2e-16$	-0.125
MODERATE	0.12	0.003	38.8	$< 2e-16$	0.118

Supplementary Table 4: Proportion of Ancestries by Region in Memphis TN and Surrounding Areas used to construct Fig1C. Center, East, South and West M refer to different regions in Memphis, TN.

Region	AFR	AMR	EUR	EUR-AFR	EUR-AMR	EAS	EUR-EAS	Multiway
Center M, TN	0.3847	0.0013	0.1835	0.3099	0.013471	0.00013	0.0001	0.1067
East M, TN	0.1626	0	0.6160	0.1626	0.0158	0	0	0.0427
South M, TN	0.1046	0.0005	0.6715	0.1494	0.0220	0	0	0.0516
West M, TN	0.2891	0.0011	0.4409	0.1996	0.0142	0	0	0.0547
Tupelo, MS	0.0776	0.0016	0.7589	0.1084	0.0129	0	0	0.0404
Jonesboro, AR	0.0822	0	0.7160	0.1359	0.0229	0	0	0.0426
Jackson, TN	0.0671	0	0.8301	0.0612	0.0297	0	0	0.0118
