# Supplementary material for: Insights from the Biorepository and Integrative Genomics pediatric resource
Source: Nat Commun. 2025 May 22;16:4750. doi: 10.1038/s41467-025-59375-0 (PMC12098674; doi:10.1038/s41467-025-59375-0)
Supplement: Supplementary file 2 — Reporting Summary [file 41467_2025_59375_MOESM2_ESM.pdf]

nature portfolio

Corresponding author(s): Vincenza Colonna

Last updated by author(s): Apr 2, 2025

# Reporting Summary

Nature Portfolio wishes to improve the reproducibility of the work that we publish. This form provides structure for consistency and transparency in reporting. For further information on Nature Portfolio policies, see our Editorial Policies and the Editorial Policy Checklist.

## Statistics

For all statistical analyses, confirm that the following items are present in the figure legend, table legend, main text, or Methods section.

| n/a | Confirmed | |
|---|---|---|
| ☐ | ☒ | The exact sample size (*n*) for each experimental group/condition, given as a discrete number and unit of measurement |
| ☐ | ☒ | A statement on whether measurements were taken from distinct samples or whether the same sample was measured repeatedly |
| ☐ | ☒ | The statistical test(s) used AND whether they are one- or two-sided<br>*Only common tests should be described solely by name; describe more complex techniques in the Methods section.* |
| ☒ | ☐ | A description of all covariates tested |
| ☒ | ☐ | A description of any assumptions or corrections, such as tests of normality and adjustment for multiple comparisons |
| ☐ | ☒ | A full description of the statistical parameters including central tendency (e.g. means) or other basic estimates (e.g. regression coefficient) AND variation (e.g. standard deviation) or associated estimates of uncertainty (e.g. confidence intervals) |
| ☐ | ☒ | For null hypothesis testing, the test statistic (e.g. *F*, *t*, *r*) with confidence intervals, effect sizes, degrees of freedom and *P* value noted<br>*Give P values as exact values whenever suitable.* |
| ☒ | ☐ | For Bayesian analysis, information on the choice of priors and Markov chain Monte Carlo settings |
| ☒ | ☐ | For hierarchical and complex designs, identification of the appropriate level for tests and full reporting of outcomes |
| ☒ | ☐ | Estimates of effect sizes (e.g. Cohen's *d*, Pearson's *r*), indicating how they were calculated |

*Our web collection on statistics for biologists contains articles on many of the points above.*

## Software and code

Policy information about availability of computer code

| | |
|---|---|
| Data collection | Data collection is managed through the Laboratory Information Management System (LIMS). |
| Data analysis | All software used for data analysis is open source and includes custom R, and Python scripting. A complete list of software is available here https://github.com/SilviaBuonaiuto/BIG |

For manuscripts utilizing custom algorithms or software that are central to the research but not yet described in published literature, software must be made available to editors and reviewers. We strongly encourage code deposition in a community repository (e.g. GitHub). See the Nature Portfolio guidelines for submitting code & software for further information.

## Data

Policy information about availability of data

All manuscripts must include a data availability statement. This statement should provide the following information, where applicable:

- Accession codes, unique identifiers, or web links for publicly available datasets
- A description of any restrictions on data availability
- For clinical datasets or third party data, please ensure that the statement adheres to our policy

The BIG data presented here is potentially identifiable human data, and therefore its availability is somewhat restricted. However, we strongly support data availability in general. Data used for this study can be shared after University of Tennessee Health Science Center institutional IRB and BIG Research Oversight Committee review and approval https://uthsc.edu/cbmi/big/. Please contact the authors for further information.

# Research involving human participants, their data, or biological material

Policy information about studies with human participants or human data. See also policy information about sex, gender (identity/presentation), and sexual orientation and race, ethnicity and racism.

| | |
|---|---|
| Reporting on sex and gender | We do not use sex or gender classification |
| Reporting on race, ethnicity, or other socially relevant groupings | Race is self-reported by enrolled patients at the time of admission to the hospital. The admission staff select the race code from a drop-down list of possible race categories according to HL7 standards for race and ethnicity https://hl7-definition.caristix.com/v2/HL7v2.5/Tables/0005. |
| Population characteristics | Our study population ranges in age from 0 to 98 years, with genotypic information derived from exome sequencing and genotype by sequencing |
| Recruitment | The BIG Initiative, based in Memphis, TN, spans several healthcare institutions and regions to support diverse genomic research. |
| Ethics oversight | University of Tennessee Health Science Center Institutional Review Board IRB number: 23-09204-NHSR |

Note that full information on the approval of the study protocol must also be provided in the manuscript.

# Field-specific reporting

Please select the one below that is the best fit for your research. If you are not sure, read the appropriate sections before making your selection.

- [x] Life sciences
- [ ] Behavioural & social sciences
- [ ] Ecological, evolutionary & environmental sciences

For a reference copy of the document with all sections, see nature.com/documents/nr-reporting-summary-flat.pdf

# Life sciences study design

All studies must disclose on these points even when the disclosure is negative.

| | |
|---|---|
| Sample size | 13,152 |
| Data exclusions | No data were excluded from this analysis |
| Replication | This was not relevant to our study |
| Randomization | We performed supervised ancestry deconvolution of the genetic data to infer the genetic ancestry of participants. While we observe a clear,uninterrupted cline of ancestry, we subdivided the data set into seven ancestry groups to account for admixture and further characterize our cohort. |
| Blinding | Blinding is not relevant for our study which is based on classification of individuals into ancestry groups |

# Reporting for specific materials, systems and methods

We require information from authors about some types of materials, experimental systems and methods used in many studies. Here, indicate whether each material, system or method listed is relevant to your study. If you are not sure if a list item applies to your research, read the appropriate section before selecting a response.

## Materials & experimental systems

| n/a | Involved in the study | |
|---|---|---|
| x | | Antibodies |
| x | | Eukaryotic cell lines |
| x | | Palaeontology and archaeology |
| x | | Animals and other organisms |
| | x | Clinical data |
| x | | Dual use research of concern |
| x | | Plants |

## Methods

| n/a | Involved in the study | |
|---|---|---|
| x | | ChIP-seq |
| x | | Flow cytometry |
| x | | MRI-based neuroimaging |

## Clinical data

Policy information about clinical studies

All manuscripts should comply with the ICMJE guidelines for publication of clinical research and a completed CONSORT checklist must be included with all submissions.

| | |
|---|---|
| Clinical trial registration | The study does not involve clinical trials |
| Study protocol | University of Tennessee Health Science Center Institutional Review Board IRB number: 23-09204-NHSR |
| Data collection | *Describe the settings and locales of data collection, noting the time periods of recruitment and data collection.* |
| Outcomes | This study does not involve primary and/or secondary outcomes measures |

## Plants

| | |
|---|---|
| Seed stocks | *Report on the source of all seed stocks or other plant material used. If applicable, state the seed stock centre and catalogue number. If plant specimens were collected from the field, describe the collection location, date and sampling procedures.* |
| Novel plant genotypes | *Describe the methods by which all novel plant genotypes were produced. This includes those generated by transgenic approaches, gene editing, chemical/radiation-based mutagenesis and hybridization. For transgenic lines, describe the transformation method, the number of independent lines analyzed and the generation upon which experiments were performed. For gene-edited lines, describe the editor used, the endogenous sequence targeted for editing, the targeting guide RNA sequence (if applicable) and how the editor was applied.* |
| Authentication | *Describe any authentication procedures for each seed stock used or novel genotype generated. Describe any experiments used to assess the effect of a mutation and, where applicable, how potential secondary effects (e.g. second site T-DNA insertions, mosiacism, off-target gene editing) were examined.* |
